# Activation of Glycine and Extrasynaptic GABA_A_ Receptors by Taurine on the Substantia Gelatinosa Neurons of the Trigeminal Subnucleus Caudalis

**DOI:** 10.1155/2013/740581

**Published:** 2013-11-28

**Authors:** Thi Thanh Hoang Nguyen, Janardhan Prasad Bhattarai, Soo Joung Park, Seong Kyu Han

**Affiliations:** Department of Oral Physiology & Institute of Oral Bioscience, School of Dentistry, Chonbuk National University, Jeonju 561-756, Republic of Korea

## Abstract

The substantia gelatinosa (SG) of the trigeminal subnucleus caudalis (Vc) has been known for the processing and transmission of orofacial nociceptive information. Taurine, one of the most plentiful free amino-acids in humans, has proved to be involved in pain modulation. In this study, using whole-cell patch clamp technique, we investigated the direct membrane effects of taurine and the action mechanism behind taurine-mediated responses on the SG neurons of the Vc. Taurine showed non-desensitizing and repeatable membrane depolarizations and inward currents which remained in the presence of amino-acid receptors blocking cocktail (AARBC) with tetrodotoxin, indicating that taurine acts directly on the postsynaptic SG neurons. Further, application of taurine at different doses (10 **μ**M to 3 mM) showed a concentration dependent depolarizations and inward currents with the EC_50_ of 84.3 **μ**M and 723 **μ**M, respectively. Taurine-mediated responses were partially blocked by picrotoxin (50 **μ**M) and almost completely blocked by strychnine (2 **μ**M), suggesting that taurine-mediated responses are via glycine receptor (GlyR) activation. In addition, taurine (1 mM) activated extrasynaptic GABA_A_ receptor (GABA_A_R)-mediated currents. Taken together, our results indicate that taurine can be a target molecule for orofacial pain modulation through the activation of GlyRs and/or extrasynaptic GABA_A_Rs on the SG neurons.

## 1. Introduction

Taurine (2-amino-ethane sulfonic acid) is one of the most plentiful free amino-acids in humans [1, 2]. In the human body, taurine is distributed with high concentration in various tissues that are excitable and/or prone to generate free radicals in retina, white blood cells, platelets, central nervous system (CNS), heart, skeletal muscles, spleen, and liver [3]. In physiological condition, taurine is accumulated in brain cells at concentration of 5–70 mM [4, 5] and is released in high amounts under various pathological conditions such as anoxaemia or ischemia and seizure [6–8]. Since its first discovery in 1827, a number of studies have been done to find out the various physiological functions and the significance of taurine. It has been reported that taurine has various functions including bile acid production [9–12], antiarrhythmic effects [13–15], and oxidant scavenging effects [16]. In central nervous system, taurine has also been reported to modulate calcium homeostasis [17, 18], neuronal excitabilities [19, 20], and excitotoxic cell death [21, 22].

The pain transmission from the orofacial region to the trigeminal subnucleus caudalis (Vc) is responsible by the first-order neurons via small-diameter primary afferents including myelinated A*δ*- and unmyelinated C-fibers [23, 24], which innervate in lamina I and in much of lamina II of the Vc [25, 26]. The lamina II called substantia gelatinosa (SG), therefore, is thought to be a key site in the processing of orofacial nociceptive information [27, 28]. The majority of neurons in the SG are local interneurons [29]. A substantial number of these interneurons contain gamma-aminobutyric acid (GABA) and glycine which are often colocalized in the same cell [30, 31]. As one of the main inhibitory neurotransmitters in the central nervous system, GABA and glycine have pivotal roles in the modulation of nociception [32–35].

A number of studies have shown that taurine is involved in pain modulation. For example, systemic and intrathecal administration of taurine induced the antinociceptive effects to inhibit the intensity of caudally-directed biting, scratching, and paw licking behaviors by chemical agent and by the hot-plate test at acute pain tests in mouse [36, 37]. It has been reported that dietary supplementation with taurine suppresses hyperalgesia in streptozotocin-induced diabetic rats and autotomy behavior in genetically selected Sabra strain rats [38]. In addition, Lee et al. showed that taurine is released from neurons in the upper dorsal horn layers which are known to conduct nociceptive input [39]. These previous reports have strongly suggested that taurine can modulate nociceptive information. Similarly, Bereiter et al. reported that there was an elevation of taurine after mustard oil (a chemical irritant) injection through the skin into the temporomandibular joint region in rats [40]. However, the action mechanism of taurine on the SG neurons which are involved in orofacial pain modulation has not been fully understood. In this study, therefore, we used the whole-cell patch clamp technique to investigate the action mechanism of taurine on the SG neurons of the Vc.

## 2. Materials and Methods

### 2.1. Animals

All experiments on living animals were ratified by Chonbuk University Animal Welfare and Ethics Committee. Immature male and female ICR mice used in the present study were housed under 12-h light : 12-h dark cycles (lights on at 07:00 h) with access to food and water *ad libitum*.

### 2.2. Brain Slice Preparation

Brain slice preparation was similar to the work done by Park et al. [41]. Briefly, the juvenile ICR mice (5-20 postnatal days) were decapitated and their brains were excised quickly, immersed in ice-cold bicarbonate-buffered artificial cerebrospinal fluid (ACSF) with the following chemical composition (in mM): 126 NaCl, 2.5 KCl, 2.4 CaCl_2_, 1.2 MgCl_2_, 11 D-glucose, 1.4 NaH_2_PO_4_, and 25 NaHCO_3_ (pH 7.3~7.4, bubbled with 95% O_2_ and 5% CO_2_). The trigeminal subnucleus caudalis segment was dissected, supported with a 4% agar block, and glued with cyanoacrylate to the chilled stage of a vibratome (Microm, Walldorf, Germany). Coronal slices (150 *μ*m in thickness, obtained 1-2 mm from the obex, the most rostral part of Vc) were prepared in ice-cold ACSF using the vibratome. The slices were kept in oxygenated ACSF at room temperature for at least 1 h before electrophysiological recording.

### 2.3. Electrophysiological Procedures and Data Analysis

The slices were transferred into a recording chamber, completely submerged, and continuously superfused with carboxygenated ACSF at a rate of 4-5 mL/min. The slices were viewed with an upright microscope (BX51W1, Olympus, Tokyo, Japan) with Nomarski differential interference contrast optics. The SG (lamina II) was clearly identified as a translucent band, just medial to the spinal trigeminal tract and traveled along the lateral edge of the slice. The patch pipettes were pulled from thin-wall borosilicate glass-capillary tubing (PG52154-4, WPI, Sarasota, USA) on a Flaming/Brown, puller (P-97, Sutter Instruments Co., Novato, CA). The pipette solution was passed through a disposable 0.22 *μ*m filter and contained the following composition (in mM): 140 KCl, 1 CaCl_2_, 1 MgCl_2_, 10 HEPES, 4 MgATP, and 10 EGTA (pH 7.3 with KOH). In this study, we used high chloride pipette solution to amplify the chloride mediated conductance. The resistance between the recording electrode filled with pipette solution and the reference electrode was 4–6 MΩ. After a gigaohm seal was formed with SG neuron, the cell membrane patch was ruptured by negative pressure, and then the whole-cell patch clamp recording was performed using an Axopatch 200B (Axon Instruments, Union City, CA). The changes in membrane potentials and membrane currents were sampled online using a Digidata 1322A interface (Axon Instruments) connected to a desktop PC. The signals were filtered (2 kHz, Bessel Filter of Axopatch 200B) before digitizing at a rate of 1 kHz. The holding current was not adjusted during the experiment and was set at 0 pA in current clamp mode. The root mean square (RMS) noises were measured in 50 ms epochs of traces lacking postsynaptic currents (PSCs), in periods of control ACSF and in the presence of strychnine and strychnine + taurine 100 *μ*M (*n* = 50 epochs in each case). The mean holding current changes within the control and treated period were calculated as the mean of peak-to-peak amplitude of individual points within each period. The acquisition and subsequent analysis of the acquired data were performed using Clampex9 software (Axon Instruments, USA). The traces were plotted using Origin7 software (MicroCal Software, Northampton, USA). All recordings were made at room temperature.

### 2.4. Drugs

The drugs used in the present study were taurine, strychnine, gabazine, picrotoxin, bicuculline (purchased from Sigma, USA), and tetrodotoxin (TTX) (from Tocris, UK). Stocks of all drugs were made according to their solubility in DMSO and in distilled water. Stocks were diluted (usually 1,000 times) to the desired final concentrations in ACSF immediately before use and were applied by bath application (4 mL/min).

### 2.5. Statistics

All values were expressed as the mean ± S.E.M. A paired *t*-test and one way ANOVA test were used to examine the difference. Statistical significance was defined as *P* < 0.05.

## 3. Results

Whole cell current and voltage clamp recordings were obtained from 98 SG neurons from juvenile mice postnatal day ranging from day 5 to day 20. A series of experiments were designed to evaluate the effects of taurine on SG neurons. The mean resting membrane potential of SG neurons tested in current clamp mode was −59.4 ± 1.61 mV (*n* = 25).

### 3.1. Taurine Induces Nondesensitizing Membrane Potential and Holding Current Changes on SG Neuron

**Figure 1:**
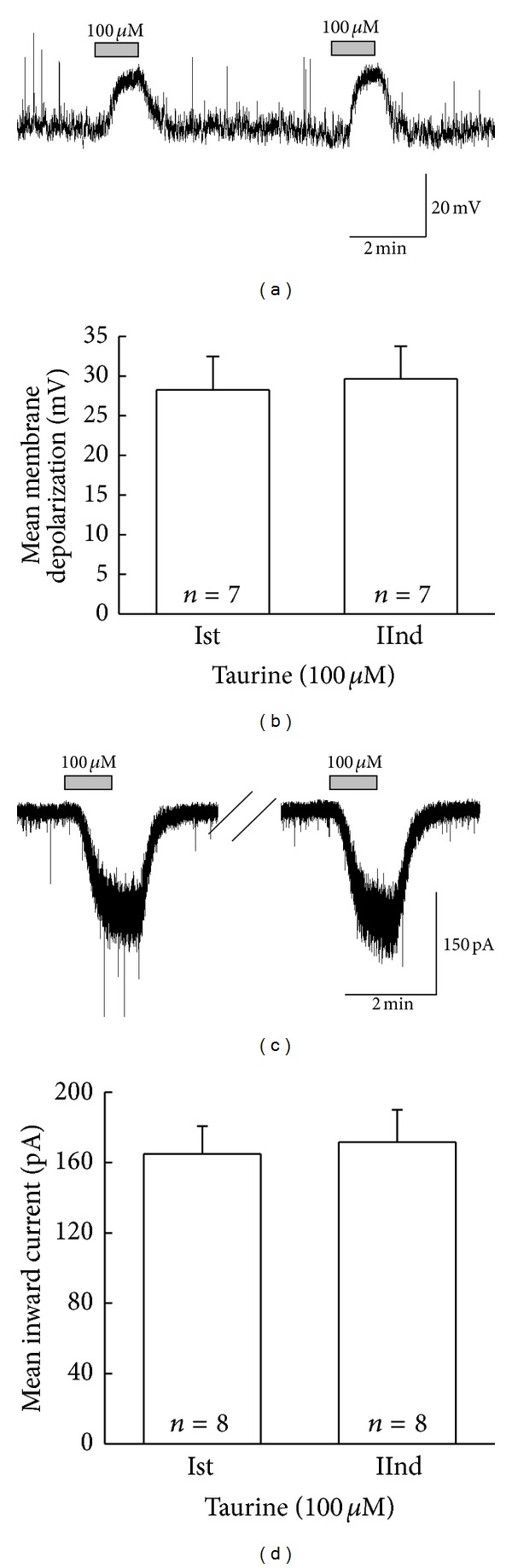
Repeated responses by the successive application of taurine on SG neurons. (a), (c) The representative traces show the repeatable membrane depolarization and repeated inward current induced by taurine (100 *μ*M). (b), (d) Bar graphs illustrate the comparison of the mean membrane potential and inward current changes by the repeated application of taurine (100 *μ*M) (*P* > 0.05).

In current and voltage clamp mode, taurine (100 *μ*M) was applied repeatedly at 5-minute time intervals to determine if the SG neurons were desensitized by successive application. In 7 SG neurons tested in current clamp mode, taurine (100 *μ*M) induced repeated membrane depolarizations (Figure 1(a)). When taurine was successively applied, the mean membrane potential change (29.7 ± 4.12 mV) by the second application was similar to that of the first application (28.3 ± 4.20 mV, *n* = 7, *P* > 0.05, Figure 1(b)). Similarly, in voltage clamp mode at holding potential of −60 mV, taurine (100 *μ*M) induced repeated inward currents (Figure 1(c)). When taurine was successively applied, the mean inward current (−172 ± 18.3 pA) by the second application was similar to that of the first application (−165 ± 15.9 pA, *n* = 8, *P* > 0.05, Figure 1(d)). These results indicate that SG neurons are not desensitized by the successively applied taurine that induces inhibitory depolarizing potentials or inward currents, respectively, at current clamp or voltage clamp mode. The mean relative membrane depolarization and the mean relative inward current of the second application were 1.06 ± 0.03 (*n* = 7) and 1.03 ± 0.04 (*n* = 8), respectively.

### 3.2. Postsynaptic Action of Taurine on SG Neurons

To investigate whether taurine affects SG neuronal activities via action potential mediated presynaptic release, the effects of taurine were examined in the presence of tetrodotoxin (TTX), a voltage sensitive Na^+^ channel blocker in current and voltage clamp mode. Taurine (100 *μ*M) induced membrane depolarization and when TTX (0.5 *μ*M) was applied, spontaneous action potentials were rapidly abolished. However, TTX did not affect the taurine-induced membrane depolarization. The mean membrane potential change (26.7 ± 4.60 mV, *n* = 7) in the presence of TTX 0.5 *μ*M was similar to that of taurine alone (28.4 ± 3.91 mV, *n* = 7, *P* > 0.05). Further, in voltage clamp experiment, the taurine-mediated inward current was not blocked by TTX. The mean inward current change (155 ± 54.6 pA, *n* = 3) in the presence of TTX was similar to that of taurine alone (162 ± 80.5 mV, *n* = 7, *P* > 0.05) (figure not shown). These results indicate that taurine-induced responses were not mediated via any action potential dependent presynaptic action on the SG neurons.

**Figure 2:**
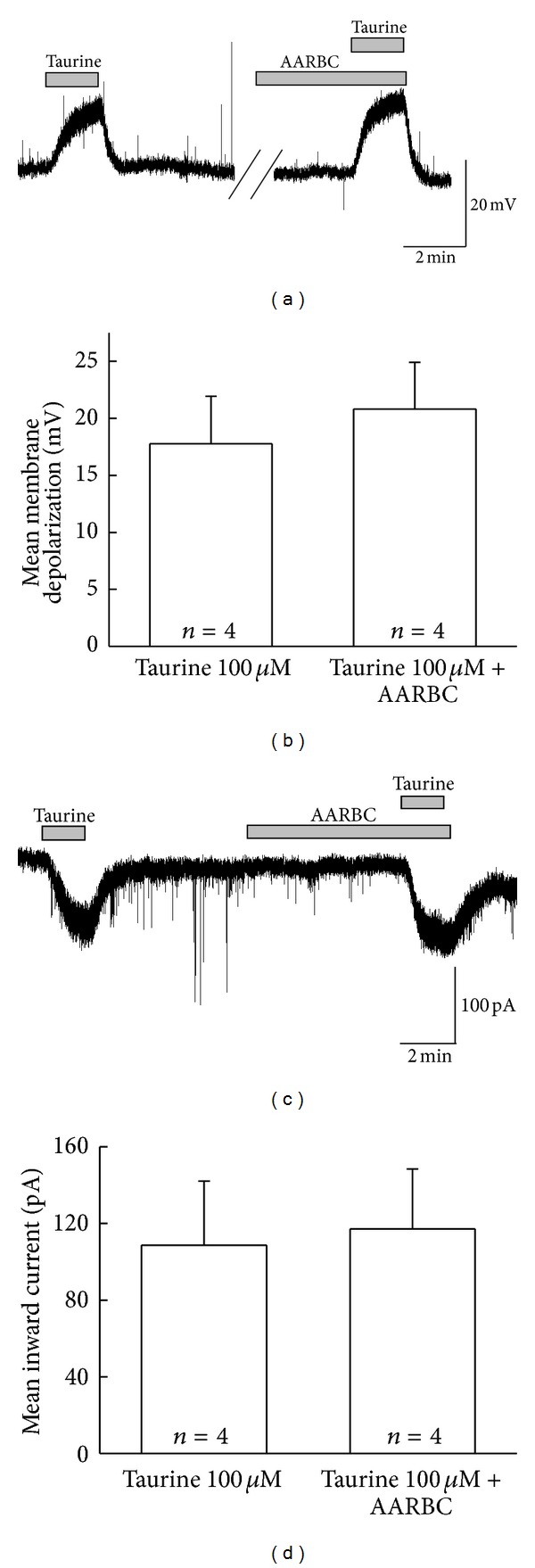
Taurine-induced membrane depolarizations and taurine-induced currents are mediated by postsynaptic SG neurons. (a), (c) The representative traces showing membrane depolarization and inward current induced by taurine (100 *μ*M) alone and taurine in the presence of AARBC. (b), (d) Bar graphs showing the comparisons of mean relative membrane depolarization and mean inward current by the taurine alone and taurine in the presence of AARBC (*P* > 0.05).

Further, we used amino-acid receptors blocking cocktail (AARBC) (6-cyano-7-nitroquinoxaline-2, 3-dione (CNQX) 10 *μ*M and (2R)-amino-5-phosphonovaleric acid (AP5) 20 *μ*M, gabazine 3 *μ*M along with tetrodotoxin (TTX) 0.5 *μ*M) to find out if taurine affects SG neuronal activities directly on the postsynaptic site. As shown in Figures 2(a) and 2(c), there were no significant differences between the responses induced by taurine alone and in the presence of AARBC. The amplitude of mean membrane depolarization induced by taurine alone (17.8 ± 4.16 mV, *n* = 4) was nearly similar to that of in the presence of AARBC (20.8 ± 4.09 mV, *n* = 4, *P* > 0.05, Figure 2(b)). Similarly, taurine-evoked mean inward currents in taurine alone and in the presence of AARBC were also almost equal (109 ± 33.4 pA and 117 ± 31.3 pA, resp., *n* = 4, *P* > 0.05, Figure 2(d)). These results put forth that taurine-mediated inward currents and depolarizations were purely postsynaptic events.

**Figure 3:**
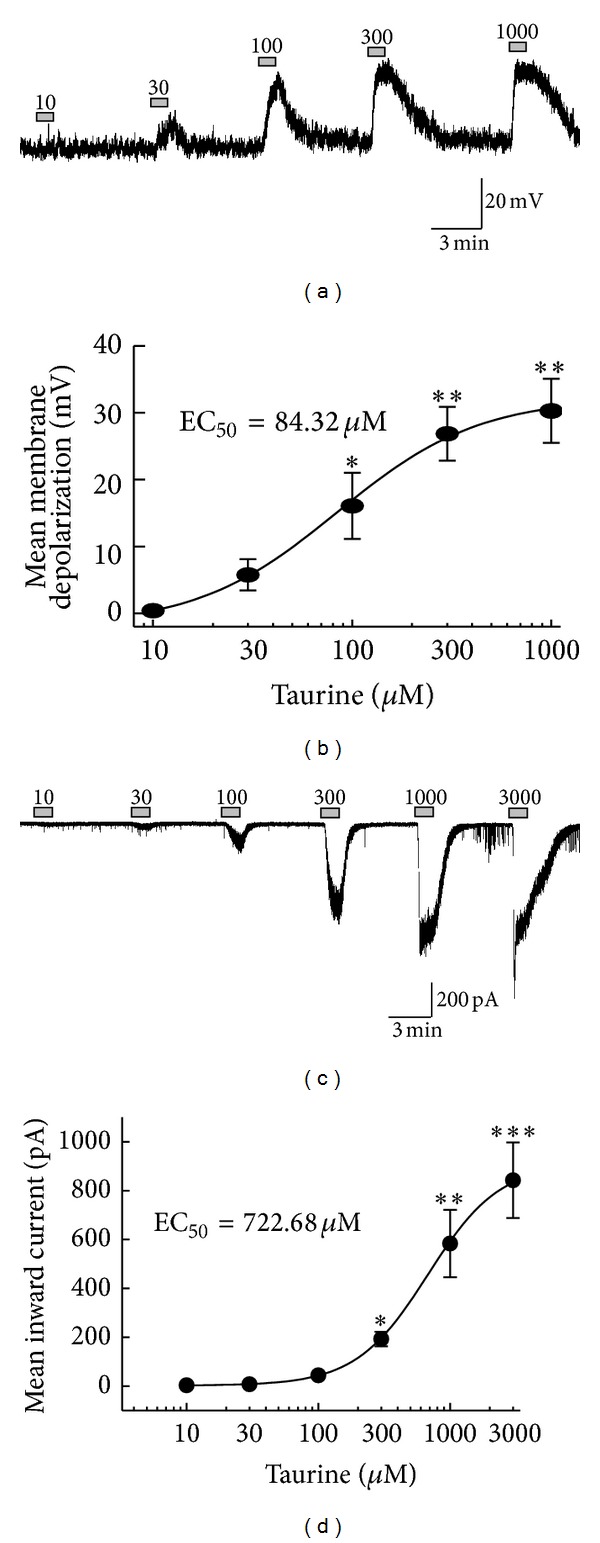
Concentration-response relationship. (a), (c) Representative traces of SG neurons showing the changes of membrane depolarizations and inward currents to different doses of taurine (10, 30, 100, 300, 1,000, 3,000 *μ*M). (b), (d) Curve figures showing the mean membrane potentials and the mean inward currents change which correspond with the concentration changes (*P < 0.05, **P < 0.01, ***P < 0.001, one-way ANOVA, Scheffe's post hoc test).

Taurine-induced membrane depolarizations and inward currents were examined at different concentrations ranging from 10 to 3,000 *μ*M. Figures 3(a) and 3(c) show the representative traces indicating the clear concentration dependency by taurine applications. Taurine-induced membrane depolarizations and inward currents were bigger at higher concentrations.  Figure 3(b) illustrates the mean membrane depolarization changes by taurine at different concentrations (10 *μ*M: 0.38 ± 0.15 mV, 30 *μ*M: 5.74 ± 2.33 mV, 100 *μ*M: 16.1 ± 4.95 mV, 300 *μ*M: 26.9 ± 4.03 mV, 1,000 *μ*M: 30.3 ± 4.80 mV, *n* = 7) with an EC_50_ of 84.3 *μ*M. Similarly, there was an increase of mean inward currents following the rise of concentration in voltage clamp mode as well (10 *μ*M: 2.88 ± 0.81 pA, 30 *μ*M: 7.06 ± 2.46 pA, 100 *μ*M: 43.9 ± 5.27 pA, 300 *μ*M: 192 ± 29.9 pA, 1,000 *μ*M: 583 ± 138 pA, 3,000 *μ*M: 842 ± 155 pA, *n* = 8) with an EC_50_ of 723 *μ*M. The values of EC_50_ were estimated by curve fitting using Origin software. This discrepancy of EC_50_ values between voltage and current clamp may be explained due to the activation of certain voltage-sensitive ion channels in current clamp mode. These concentration dependent responses also support that taurine acts on the postsynaptic site of SG neurons directly.

### 3.3. Taurine Activates Glycine Receptors on SG Neurons

**Figure 4:**
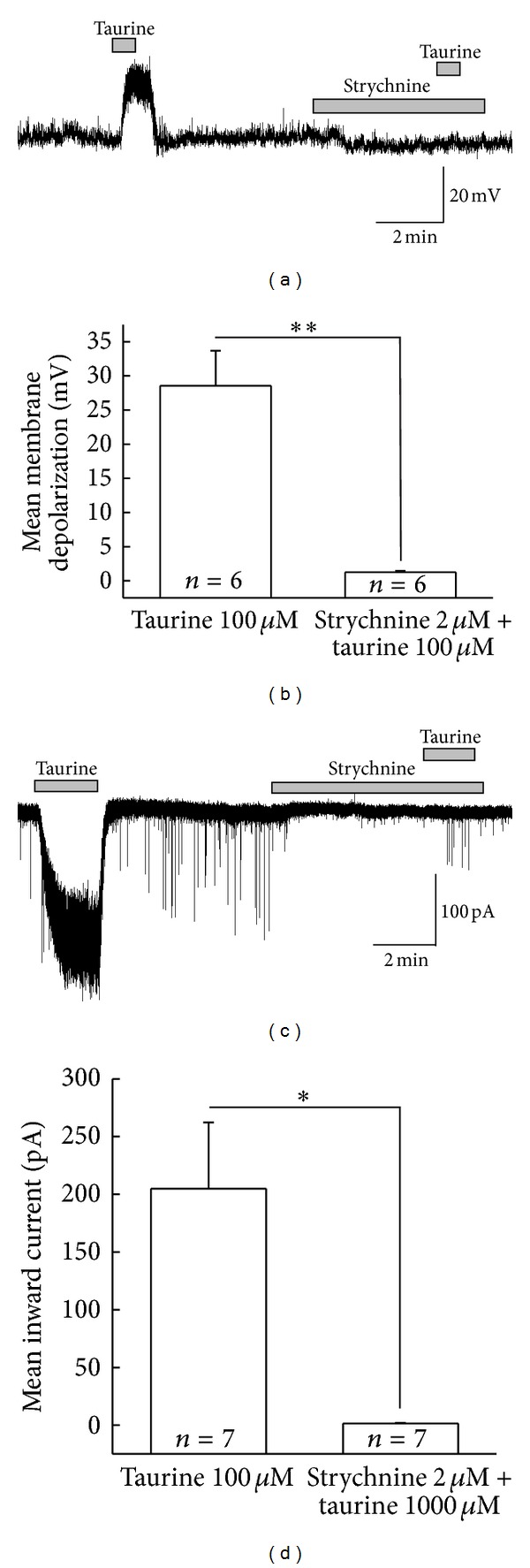
Inhibition of taurine-induced membrane depolarization and inward current by strychnine on SG neurons of Vc. (a), (c) Representative traces showing the taurine-induced membrane depolarization and taurine-induced inward current were blocked by strychnine (2 *μ*M), a glycine receptor (GlyR) antagonist. (b), (d) Bar graphs showing the comparisons of mean relative membrane potential and inward current changed by the taurine alone and in the presence of strychnine (*P < 0.05, **P < 0.01).

It has been reported that taurine can activate GlyRs in ventromedial hypothalamic neurons [42], supraoptic magnocellular neurons [43], cultured neurons of auditory cortex [44], and anteroventral cochlear nucleus neurons [45]. To check whether taurine-induced membrane depolarizations and inward currents on the SG neurons of the Vc were mediated by GlyR activation, strychnine, a selective GlyR antagonist was used. As shown in Figures 4(a) and 4(c), taurine-induced membrane depolarization and current were almost blocked by strychnine (2 *μ*M). The mean membrane depolarizations induced by the application of taurine in the absence and presence of strychnine were 28.5 ± 5.14 mV and 1.25 ± 0.19 mV, respectively (*n* = 6, Figure 4(b), *P* < 0.01). In addition, the mean inward current induced by taurine (205 ± 57.4 pA) was eliminated by the simultaneous application with strychnine (1.38 ± 0.58 pA) (*n* = 7, Figure 4(d), *P* < 0.05).

### 3.4. Taurine-Induced Actions Were Mediated via GlyRs and Extrasynaptic GABA_A_ Receptors

It has been reported that taurine can activate GABA_A_ receptors (GABA_A_Rs) in various regions such as main olfactory bulb [46, 47], in the hippocampal CA1 area [48], and in anteroventral cochlear nucleus neurons [45]. As gabazine is well known to block synaptic GABA_A_Rs at lower concentration [49] as well as extrasynaptic GABA_A_Rs at higher concentration [50], taurine was applied in the presence of gabazine (3 *μ*M).

**Figure 5:**
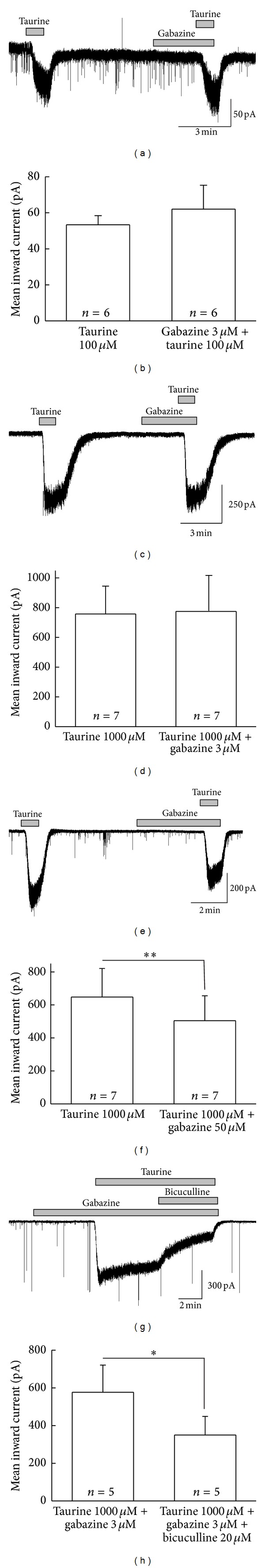
Taurine-induced inward current is only sensitive to gabazine at high concentration on SG neurons. (a), (c), (e) The representative traces showing the responses to taurine (100 *μ*M and 1,000 *μ*M) were not affected by gabazine 3 *μ*M but were affected by gabazine 50 *μ*M. (b), (d), (f) Bar graphs showing no significant difference about mean inward currents between the application taurine alone and taurine in the presence of gabazine 3 *μ*M (*P* > 0.05), but there was a considerable change in the presence of gabazine 50 *μ*M (*P* < 0.01). (g) The representative trace showing the inhibition of taurine-induced inward current in the presence of gabazine by GABA_A_ broad antagonist bicuculline (20 *μ*M). (h) The bar graph showing the mean inward current induced by taurine 1,000 *μ*M in the presence of gabazine 3 *μ*M and the mean remaining response after being blocked by bicuculline 20 *μ*M (*P* < 0.05). Holding potential was −60 mV.

The currents activated by taurine at 100 *μ*M and 1,000 *μ*M were not affected by 3 *μ*M gabazine (Figures 5(a) and 5(c)). Figures 5(b) and 5(d) compare the changes in inward currents between taurine alone (with two different concentrations 100 *μ*M and 1,000 *μ*M (53.4 ± 5.06 pA and 758 ± 187 pA, resp.)) and taurine in the presence of gabazine 3 *μ*M (62.1 ± 13.3 pA and 774 ± 235 pA, resp.). Therefore, at these concentrations, GABA_A_Rs are not affected by taurine. On the other hand, to identify whether taurine can act on extrasynaptic GABA_A_Rs on SG neurons, the concentration of gabazine was increased to 50 *μ*M (Figures 5(e) and 5(f)). The taurine-induced current was inhibited by gabazine at high concentration (Figure 5(e)). Specifically, the mean inward current induced by taurine 1,000 *μ*M (648 ± 173 pA) was reduced to 504 ± 151 pA in the presence of gabazine 50 *μ*M (Figure 5(f), *P* < 0.01). Further additional experiments in the presence of gabazine and bicuculline were conducted to figure out the activation of extrasynaptic GABA_A_Rs current by 1,000 *μ*M taurine, and as expected, bicuculline blocked the taurine-induced inward current in the presence of gabazine (Figures 5(g) and 5(h), *P* < 0.05).

**Figure 6:**
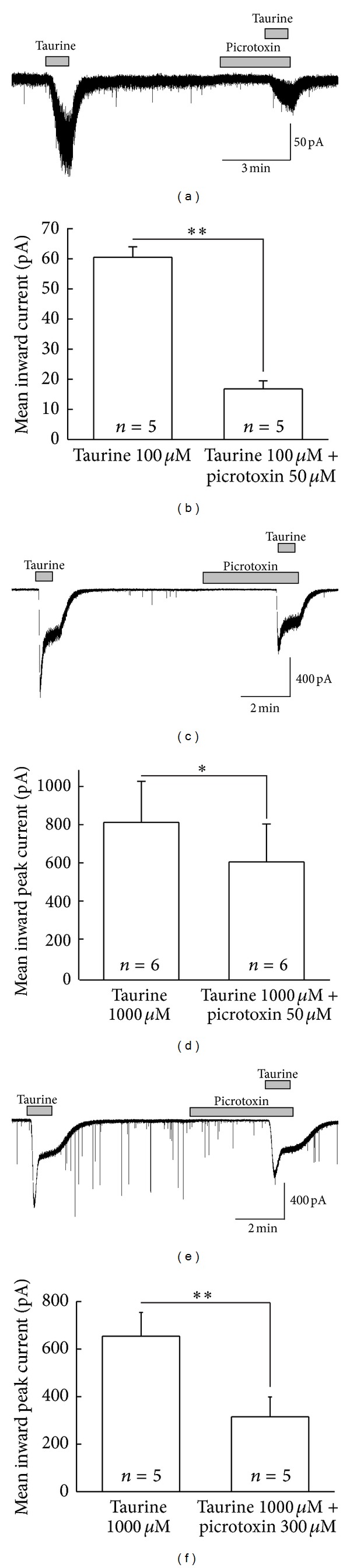
Taurine-induced inward current is sensitive to picrotoxin on SG neurons. (a), (c), (e) The representative traces showing currents evoked by 100 *μ*M and 1,000 *μ*M taurine were blocked by picrotoxin 50 *μ*M and 300 *μ*M. (b), (d), (f) Comparison of mean inward current changed by taurine alone with taurine in the presence of picrotoxin (*P < 0.05, **P < 0.01). Holding potential was −60 mV.

There are a plethora of studies suggesting that the GABA_A_R receptor antagonist picrotoxin also blocks extrasynaptic homomeric glycine receptors at lower concentration of 50–100 *μ*M and is used extensively to characterize the glycine receptors on neuronal populations. So, here in this study we tested taurine in the presence of picrotoxin to characterize the type GlyRs activated by taurine on SG neurons of Vc. Taurine-induced inward currents on SG neurons were blocked by picrotoxin 50 *μ*M (Figures 6(a) and 6(c)). The mean inward currents evoked by taurine 100 *μ*M and 1,000 *μ*M were significantly decreased in the presence of picrotoxin (50 *μ*M). The mean inward currents evoked by taurine 100 *μ*M and 1,000 *μ*M in absence and presence of picrotoxin were 60.5 ± 3.43 pA; 813 ± 216 pA and 16.8 ± 2.71 pA; and 605 ± 199 pA, respectively (Figures 6(b) and 6(d)). These results suggest that the SG neurons of Vc functionally express both heteromeric and homomeric GlyRs. Interestingly, it is very clear from Figures 6(b) and 6(d) that the inhibition of 1,000 *μ*M taurine-mediated response by picrotoxin (50 *μ*M) was less than that of 100 *μ*M taurine. This result can be explained considering that there might be a possibility that at higher concentration of taurine may affect extrasynaptic GABA_A_Rs. In addition, at high concentration of picrotoxin (300 *μ*M), 1,000 *μ*M taurine-induced currents were further decreased (Figures 6(e) and 6(f)), suggesting the activation of extrasynaptic GABA_A_Rs by higher concentration of taurine.

**Figure 7:**
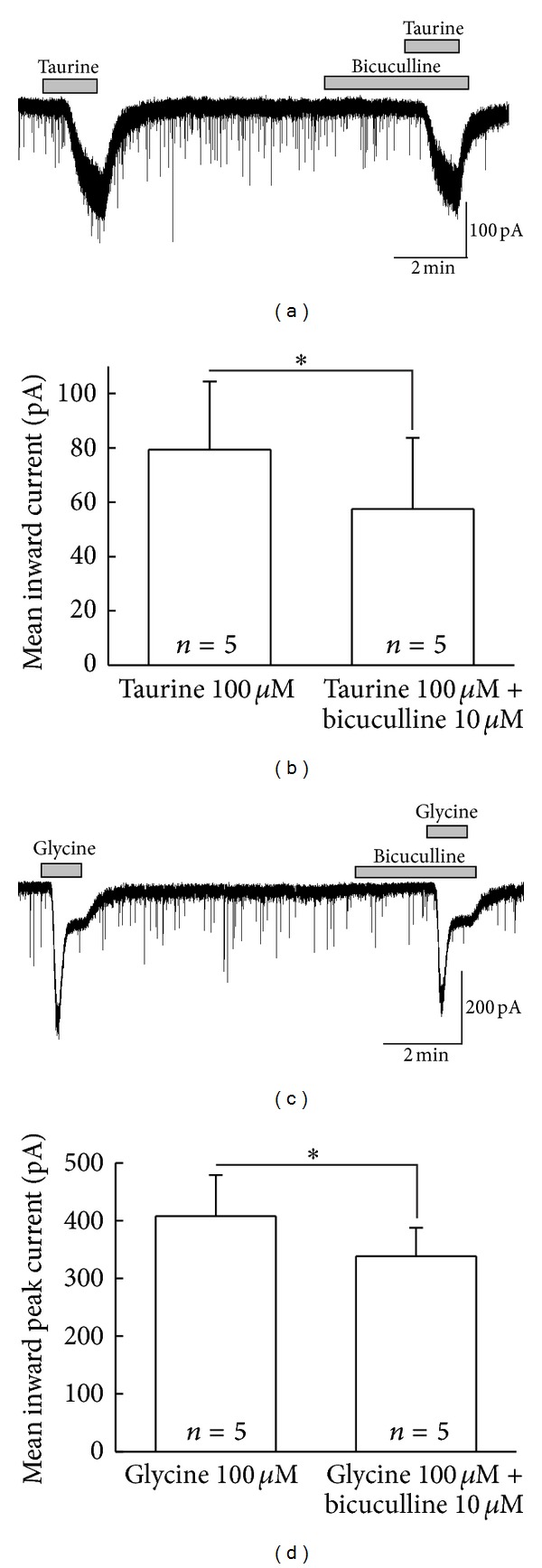
Sensitivity of taurine- and glycine-induced current to bicuculline. (a), (c) Currents activated by taurine and glycine were inhibited by bicuculline. (b), (d) The bar graphs show that mean inward currents effected by taurine and glycine were both reduced by the simultaneous application of bicuculline (*P* < 0.05) Holding potential was −60 mV.

Following this further, we also used another selective GABA_A_R antagonist, bicuculline, which follows the same pattern as picrotoxin does, that is, blockade of homomeric GlyRs [51]. We confirmed the inhibitory effect of bicuculline on taurine and glycine-mediated responses. Figures 7(a) and 7(c) show the inhibition of bicuculline on the taurine and glycine-induced currents. The mean inward currents by taurine 100 *μ*M in the absence and presence of bicuculline 10 *μ*M were 79.3 ± 25.1 pA and 57.6 ± 26.2 pA (Figure 7(b)), respectively. Whereas the mean inward currents elicited by glycine (100 *μ*M) in the absence and presence of bicuculline (10 *μ*M) were 408 ± 71.5 pA and 339 ± 48.6 pA (Figure 7(d), *n* = 5), respectively.

**Figure 8:**
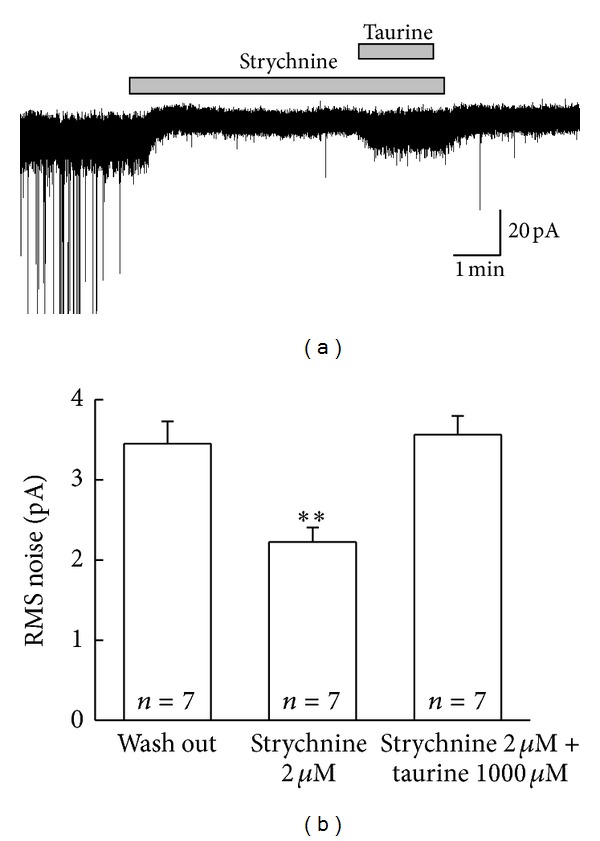
Taurine-mediated tonic conductance via extrasynaptic glycine and GABA receptors on SG neurons. (a) The representative trace illustrated that strychnine 2 *μ*M mediated an outward shift of holding current by blocking glycine-mediated neurotransmission and blocked the taurine-induced synaptic currents except GABA_A_R-mediated extrasynaptic current. (b) The bar graph showing the comparison of RMS noise in intact condition, in the presence of strychnine 2 *μ*M and in the spontaneous application of taurine 1,000 *μ*M and strychnine 2 *μ*M (**P < 0.01). Holding potential was −60 mV.

Further, in a quest to figure out the actual extrasynaptic glycine and GABA_A_ receptors mediated tonic currents by 1,000 *μ*M taurine on SG neurons, it was applied in the presence of strychnine. Strychnine dramatically blocked the synaptic currents and induced outward shift of the holding current (Figure 8(a)). Presumably, this blockade of synaptic currents were via heteromeric GlyRs, and outward shift of holding current was induced via extrasynaptic GlyRs. Moreover in the presence of strychnine, taurine (1,000 *μ*M) induced the inward current with increase in RMS noise. RMS noise in intact condition, in the presence of strychnine and in the presence of strychnine and taurine were 3.45 ± 0.28 pA, 2.23 ± 0.18 pA and 3.56 ± 0.23 pA, respectively (*n* = 7, Figure 8(b), *P* < 0.01).

## 4. Discussion

The results of this study can be summarized as follows. SG neurons were not desensitized by the application of taurine. The taurine-induced membrane depolarizations on SG neurons were mediated by postsynaptic actions. There was concentration-response relationship between taurine and SG neurons. Taurine acted as an agonist on both extrasynaptic homomeric and synaptic hetromeric GlyRs on the SG neurons. Taurine at higher concentration could affect extrasynaptic GABA_A_Rs.

Taurine has been demonstrated for its ability in modulation of synaptic transmission by activating GlyRs and/or  GABA_A_Rs. However, the physiological actions of taurine which can be upon either GlyRs or GABA_A_Rs have been also proved to depend on the specific brain region studied [46, 47]. For example, taurine activates both GABA_A_Rs and GlyRs in neurons of the supraoptic nucleus, Xenopus oocytes, and the hippocampal CA1 area [43, 48, 52] and activates only GABA_A_Rs receptors in mitral and tufted cells from the rat main olfactory bulb [47]. In addition, this activation of taurine in some brain regions is concentration-dependent. For instance, in young rat hippocampus, nucleus accumbens, and adult rat supraopic nucleus, taurine cannot only activate GlyRs at a low concentration (≤1 mM) but can activate GABA_A_Rs as well at a high concentration (≥3 mM) [43, 48, 53]. On the other hand, the findings by Song et al. in 2012 have shown that in anteroventral cochlear nucleus neurons, at low (0.1 mM) and high (1 mM) concentrations, taurine can activate both GABA_A_Rs and GlyRs [45].

In the mammalian CNS, GlyRs are formed by a combination of five distinct transmembrane protein subunits, one *β* subunit and four *α* subunit (*α*1–*α*4) [54, 55]. This composition influences in two different ways of forming functional receptors: the homomeric configuration comprising five *α* subunits and the heteromeric configuration composed of 2*α* : 3*β* subunits [55–57]. The physiological and pharmacological properties of GlyRs are dependent on the subunit combination. Picrotoxin, a GABA_A_R  antagonist, is proved as a standard tool to discriminate between homomeric and heteromeric GlyRs [58]. At low concentration of 50–100 *μ*M, picrotoxin selectively blocks homomeric GlyRs but not heteromeric receptors. In this study, to pharmacologically characterize the type of GlyRs present on SG neurons, taurine and glycine 100 *μ*M were applied in the presence of picrotoxin. The result indicate that glycine- and taurine-induced inward currents were partially blocked by picrotoxin (50 *μ*M), suggesting the presence of *α* homomeric GlyRs. However, this blockade was not complete and the unblocked remainder implies the activation of another GlyRs, likely *αβ* heteromeric GlyRs. The result in this study puts forth that taurine activates not only the synaptic hetromeric GlyRs but also the homomeric extrasynaptic GlyRs giving the tonic glycinergic inhibition on SG neurons, as established on spinal cord and hippocampal neurons [59, 60].

Another major inhibitory neurotransmitter in the CNS is GABA which mediates its most rapid effects via the ionotropic GABA_A_Rs. GABA_A_Rs which are pentameric ligand-gated ion channels consisting of diverse subunits are typically composed of two *α* and two *β* subunits together with *γ*2 subunit [61]. The difference of subunit composition influences not only the properties and function of receptors but also their distribution within the cellular membrane [62, 63]. GABA_A_ receptors, containing the *γ*2 subunit, are preferentially located in the synapse and generate “phasic” inhibitory postsynaptic currents [64]. On the other hand, in some receptors, the *δ* subunit can take the place of the *γ*2 subunit. The existence of the *δ* subunit leads to receptor expression in the extrasynaptic membrane and the activation of these receptor results in the generation of “tonically” active currents [65–68]. In the present study, inward current with increased RMS noise by taurine 1,000 *μ*M in the presence of strychnine and unaffected current in the presence of gabazine 3 *μ*M which blocks the synaptic GABA_A_Rs  suggests the activation of extrasynaptic GABA_A_Rs by taurine 1,000 *μ*M. The activation of extrasynaptic GABA_A_Rs by taurine may have important physiological and pathophysiological effects to protect neurons from toxicity under pathological conditions [22].

Glycine and GABA are known to be inhibitory neurotransmitters. Within the SG of the spinal dorsal horn, these neurotransmitters take part in the modulation of sensory input by exerting powerful inhibitory effects on spontaneous and afferent evoked activity in second-order neurons [69]. In previous studies, GABA_A_R- and GlyR-mediated conductance have been found to have inhibitory effects on orofacial nociceptive input [70]. Likewise taurine has also been shown to have inhibitory effect on other brain areas [71]. In this study, activation of glycine and GABA receptors by taurine on SG neurons has given a clear evidence that taurine behaves as an inhibitory neurotransmitter on the SG neurons of Vc. Because of this property, taurine symbolizes essential targets in descending pathways to orofacial pain.

The significant increase of taurine level in the brain under pathological conditions in response to electrical, chemical, and pain stimulation signals that taurine may play a role in neuroprotection [72–74]. With the physiological ability to activate the inhibitory neurotransmitter receptor in SG neurons, our results indicate that the influence of taurine on SG neurons may be an important modulation which has a part in the processing of orofacial nociceptive information. Further researches need to be done to ascertain the antinociceptive role of taurine to orofacial pain.
